# The association between soy intake and risk of gestational diabetes mellitus: a prospective cohort study

**DOI:** 10.1186/s12884-021-04175-9

**Published:** 2021-10-13

**Authors:** Yan Wang, Biru Luo, Jie Xiang

**Affiliations:** 1 West China School of Nursing/Key Laboratory of Birth Defects and Related Diseases of Women and Children (Sichuan University), Ministry of Education, Sichuan University, Chengdu, 610041 China; 2 Department of Nursing, West China Second University Hospital/West China School of Nursing, Sichuan University, Chengdu, 610041 China; 3 Key Laboratory of Birth Defects and Related Diseases of Women and Children (Sichuan University), Ministry of Education, Chengdu, 610041 China

**Keywords:** Gestational diabetes mellitus, Cesarean section, Soy, Prevention, Cohort study

## Abstract

**Background:**

The association between soy intake and adverse pregnancy outcomes remains unclear. The objectives of this study were to investigate the soy consumption of pregnant women in the second trimester and explore the prospective association between soy intake and the risk of adverse pregnancy outcomes.

**Methods:**

Pregnant women between 13 and 24 weeks of gestation were recruited at a women’s and children’s hospital in southwest China from June to December 2019. Dietary intakes in the middle trimester were assessed by a semi-quantitative food frequency questionnaire. Participants were divided into the insufficient group (< 40 g/day) and the control group (≥40 g/day) according to daily soy consumption. Participants were followed up until delivery. Pregnancy outcomes including gestational diabetes mellitus (GDM), cesarean section, and macrosomia were obtained. Multiple logistic regression was used to analyze the association between soy intake and risk of adverse pregnancy outcomes. Sociodemographic information, histories of diseases, and duration of physical activities were obtained and used for covariate adjustments.

**Results:**

A total of 224 participants were included in this study, of which identified 36 (16.1%) cases of GDM, and 120 (53.6%) cases of cesarean section. More than half (125, 55.8%) pregnant women consumed less soy than 40 g/day. Daily soy intake less than 40 g was associated with the increased risk of GDM (*OR* = 2.755 95%CI 1.230-6.174, *P* = 0.014) and cesarean section (*OR* = 1.792 95%CI 1.035-3.101, *P* = 0.037) without adjustment for confounders such as age, pre-pregnancy body mass index, parity, daily intake of vegetables, fruits, seafood and, nuts. After adjusting for these factors, daily soy intake of less than 40 g increased 2.116-fold risk of GDM (95%CI 1.228-7.907, *P* = 0.017), but not with the significantly increased risk of cesarean section.

**Conclusion:**

Insufficient soy intake may increase the risk of GDM, suggesting adequate soy intake may have a beneficial role in the prevention of GDM.

**Trial registration:**

Registration number: ChiCTR1900023721. Date of registration: June 9, 2019.

## Background

Soy is a kind of legume and widely used as a protein supplement in the world. Soy has various health benefits for humans because soy is a low-glycemic-index food containing low energy, dense nutrient, high fiber, and plenty of plant protein [1]. Moreover, soy contains various minerals such as calcium and kalium [2], contributing to glycemic control and glucose homeostasis [3]. Studies evaluating the correlation between soy consumption and diabetes prevention on humans show controversial results [4]. Some studies showed that soy consumption did not affect measures of glycemic control [3, 5], but many studies have demonstrated adequate intake of soy or soy products plays a beneficial role in decreasing the risk of type 2 diabetes and diabetic complications such as cardiovascular disease [6–8].

Soy intake is also associated with the health outcomes of mother and infant. A study reported soy intake was inversely related to maternal weight gain and positively associated with birth weight [9]. Additionally, studies have shown soy supplements may reduce the risk of preterm birth and maternal anemia in late gestation [10] and may protect against the adverse reproductive effects of bisphenol A including low live birth rates [11]. Some studies found that inadequate soy intake in the second trimester increased the risk of gestational diabetes mellitus (GDM) [12, 13]. An interventional study showed that soy treatment improved metabolic status in patients with GDM, including improving glucose homeostasis parameters, triglycerides, and biomarkers of oxidative stress [14]. Therefore, dietary guidelines of many countries recommend soy products as a protein supplement for pregnant women. 2020-2025 Dietary Guidelines for Americans recommended that pregnant women consumed 4-6 oz of soy products, seeds and nuts per week as a plant protein supplement [15], and 2015-2020 Dietary Guidelines for Americans recommended soy products as a protein food and fortified soy beverages as dairy alternatives [16]. People in Europe and America mainly get protein from meat. However, plants, especially soy, are an important source of dietary protein supplements for people in East and Southeast Asia, where consumption of meat is traditionally low. Therefore, the Chinese Nutrition Society recommends Chinese pregnant women consume at least 40 g of soy a day as a protein supplement, with a maximum of 60 g per day. Soy is recommended as a dietary supplement for several reasons. First, soy has been cultivated in China for a long time, forming many processing methods, which is beneficial for pregnant women to intake. Second, soy is recognized as the food with the highest protein content and is considered a substitute for the protein found in meat, eggs, and dairy products [17]. Third, compared with meat, soy is cheaper and easier to get, especially for pregnant women living in rural areas. However, previous studies showed that soy intake among Chinese pregnant women is not optimistic. Studies reported only minority pregnant women consume adequate soy or soy products [18, 19]. In recent years, much attention has been paid to dietary guidance for pregnant women. However, it remains unclear whether soy intake is valued in perinatal care.

To date, only a few studies have explored the association between soy intake and pregnancy outcomes. The relationship between soy intake and some birth outcomes such as GDM was controversial. Some studies reported a negative association between soy consumption and incidences of GDM [12, 13]. However, one study showed a positive association between intake frequency of soy milk and incidences of GDM [20]. Therefore, the objectives of this study were to investigate the soy intake of pregnant women in the second trimester and explore the association between soy intake and risk of adverse pregnancy outcomes, especially GDM.

## Methods

### Study design and participants

A prospective cohort study was performed. The study cohort was established between June and December 2019 in West China Second University Hospital, Sichuan University. Participants in this cohort were recruited from the Department of Obstetrics in this hospital using the convenience sampling method. Participants recruited into the study needed to fulfill the following criteria: i) aged between 18 and 40 years old; ii) 13-24 weeks of gestational age; iii) no serious complications such as heart disease or hypertension; iv) no pregnancy complications, such as gestational hypertension, placenta previa, etc.; v) no mental illness. Participants who missed key information such as oral glucose tolerance test (OGTT) results and pregnancy outcomes, and had pre-pregnancy diabetes were excluded.

### Data collection

The data were collected on the day when participants entered the study through a face-to-face interview in the outpatient department. A self-designed questionnaire was used to collect sociodemographic characteristics and clinical information such as age, pre-pregnancy weight, height, educational level, religion, ethnicity, occupation, present illness, history of GDM, family history of diabetes, gravidity, parity, and the number of abortions, etc. The questionnaire took about 30 mins to complete.

Data for soy intake was evaluated using the Food Frequency Questionnaire (FFQ), a Chinese version questionnaire amended by Jing [21]. FFQ recorded the frequency and intake amount of thirteen food (rice noodles, cereal, potatoes food, vegetables, fruits, livestock meat, poultry meat, seafood, eggs, milk and dairy products, soy, nuts, and oils) of participants within the most recent 1 month. The intake of thirteen kinds of food was divided into ten categories: grains, vegetables, fruits, meat, seafood, eggs, milk, soy, nuts, and oils. The participants completed the part on intake frequency. YW completed the part on the intake of food based on the food model book containing photos of kinds of food and amounts after consulting the participants. The Cronbach’s α coefficient of FFQ in this study was 0.739, suggesting good internal consistency.

Duration of physical activity was collected to adjust for its potential confounding influences on the association between soy intake and pregnancy outcomes. Data for physical activity was evaluated using Physical Activity Scale (PAS) [22], which was developed by Aadahl [23], then translated and introduced to China by Jiang [22]. This questionnaire, including nine items, used in Chinese pregnant women to collect their physical activity about the intensity, time spent, and energy expenditure of various categories. The physical activity intensity was classified into 9 categories from A to I, with MET values of 0.9, 1,1.5, 2.0, 3.0, 4,5,6, > 6 MET (1MET = oxygen consumption 3.5 ml/ (kg· min) = energy consumption 0.0167 kcal/ (kg. min)). The reliability and validity of the PAS were confirmed in a previous study [22]. The Cronbach’s α coefficient of PAS was 0.718, suggesting good internal consistency.

### Grouping

The daily intake of soy was calculated by multiplying the number of times a day by each intake amount. According to the Chinese Nutrition Guideline for pregnant women, the daily intake of soy for women in the second trimester should be at least 40 g [24]. Therefore, participants were divided into an insufficient group and a control group based on whether their daily soy consumption was 40 g or more than 40 g.

### Outcome

Data for pregnancy outcomes were collected at two time points: 1) after OGTT: all participants accepted one-step 75-g OGTT at 24-28 weeks of gestation. GDM was diagnosed if one or more blood glucose levels reached or exceeded the International Association of Diabetes Pregnancy Study Groups (IADPSG) criteria (fasting: 5.1 mmol/L; 1 h:10.0 mmol/L; 2 h: 8.5 mmol/L). The diagnostic results of GDM, OGTT results, and glycated hemoglobin values were collected; 2) after delivery: maternal outcome including pregnancy-induced hypertension status, weight gain, delivery gestational age, delivery mode, postpartum hemorrhage, preterm birth, and premature rupture of membranes (PROM), and neonatal outcome including macrosomia, birth weight, height were collected from the hospital information system.

### Statistical analysis

SPSS version 23 was used to analyze data in this study. Quantitative data of normal or skewed normal distribution were described as mean and standard deviation (*SD*). Independent t-test was used to compared the difference between the two groups. Quantitative data of non-normal distribution were described as median and interquartile range (*IQR*), and compared by Mann Whitney U test. Qualitative data were described as frequency (n) and percentage (%), and compared by chi-square test. A stepwise multivariate logistic regression analysis was composed. To adjust for the potential influences from confounding factors on the association between soy intake and pregnancy outcomes, univariate analysis was performed on sociodemographic characteristics, dietary intake, and physical activity in the two groups to identify potential confounding factors. The logistic regression model was adjusted for variables with *P*-value < 0.05 in univariate analysis. Odds ratio (*OR*) was calculated. All *P* values were two-sided, and *P*-value < 0.05 was considered statistically significant.

## Results

### Characteristics of participants

**Fig. 1:**
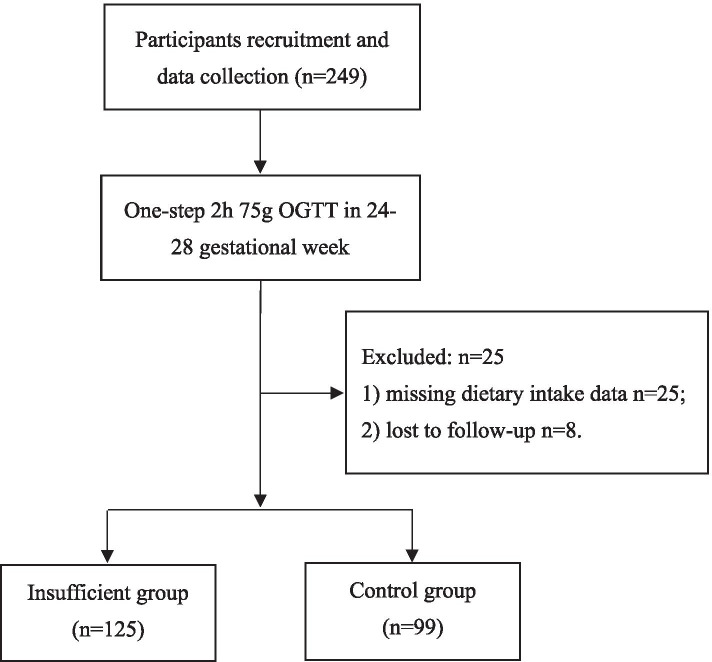
The flow chart OGTT = oral glucose tolerance test; Insufficient group: daily soy intake < 40 g; Control group: daily soy intake ≥40 g

A total of 249 pregnant women were recruited in this study. Fifteen pregnant women were excluded because of missing the data of daily soy intake. Ten women who were lost to follow-up were excluded (Fig. 1). Finally, 224 participants were included in the final analysis, of which 125 (55.8%) pregnant women had a daily intake of less than 40 g soy. The mean age of all participants was 30.35 (*SD* 3.69) years old. The number of multiparous women in the insufficient group was more than that in the control group (*χ*^*2*^ = 4.235, *P* = 0.040). There were no statistical differences in other sociodemographic characteristics and clinical characteristics between the insufficient group and the control group (Table 1).

**Table.1:** The characteristics of participants (*n* = 224)

Characteristics	All	Soy intake < 40 g/d	Soy intake ≥40 g/d	*P* value
Age (mean ± SD, year)	30.35 ± 3.69	30.78 ± 0.36	29.83 ± 3.21	0.058
Pre-pregnancy weight (mean ± SD, kg)	53.09 ± 7.30	53.06 ± 6.75	53.14 ± 7.98	0.934
Height (mean ± SD, cm)	159.84 ± 4.43	159 ± 4.17	160.37 ± 4.71	0.108
Pre-pregnancy BMI (mean ± SD, kg/m^2^)	20.78 ± 2.77	20.87 ± 2.51	20.67 ± 3.08	0.582
Fasting glucose (first trimester, mean ± SD, mmol/L)	4.34 ± 0.41	4.31 ± 0.40	4.38 ± 0.42	0.282
Gestation (mean ± SD)	2.09 (1.14)	2.10 (1.05)	2.08 (1.25)	0.885
Production (median (IQR)) ^*^	0.32 (0.52)	0.36 (0.48)	0.27 (0.57)	0.167
Abortion (median (IQR)) ^*^	0.77 (0.95)	0.73 (0.84)	0.83 (1.08)	0.471
Ethnicity n (%)				0.753
Han ethnicity	217 (96.9)	122 (97.6)	95 (96.0)	
Minority	7 (3.1)	3 (2.4)	4 (4.0)	
Occupation status n (%)				0.731
Unemployed	47 (21.0)	25 (20.3)	22 (22.2)	
Employed	175 (78.1)	98 (79.7)	77 (77.8)	
Missing	2 (0.9)	–	–	
Education level n (%)				0.965
Junior high school	3 (1.3)	2 (1.7)	1 (1.0)	
Senior high school	16 (7.1)	9 (7.8)	7 (7.3)	
College	53 (23.7)	28 (24.1)	25 (26.0)	
Bachelor and above	140 (62.5)	77 (66.4)	63 (65.6)	
Missing	12 (5.4)	–	–	
Parity n (%)				0.040
Nulliparous	154 (68.8)	79 (63.7)	75 (76.5)	
Multiparous	68 (30.4)	45 (36.3)	23 (23.5)	
Missing	2 (0.9)	–	–	
Elderly primipara (n (%), ≥35 years old)	9 (0.4)	6 (4.8)	3 (3.0)	0.743
Multiple pregnancy n (%)	12 (5.4)	6 (4.8)	6 (6.1)	0.677
PCOS n (%)	2 (0.9)	2 (1.6)	0 (0)	0.505
Pre-pregnancy diabetes n (%)	1 (0.4)	0 (0)	1 (1.0)	0.442
Family history of diabetes n (%)	29 (12.9)	16 (12.8)	13 (13.1)	0.942
History of GDM n (%)	6 (2.7)	4 (3.2)	2 (2.0)	0.587
Family history of hypertension n (%)	31 (13.8)	20 (16.0)	11 (11.1)	0.293
IVF n (%)	14 (6.3)	10 (8.0)	4 (4.0)	0.224

*Note*: *M* mean, *SD* standard deviation, *IQR* interquartile range, *BMI* body mass index, *PCOS* polycystic ovary syndrome, *GDM* gestational diabetes mellitus, *IVF* in-vitro fertilization *g/d* gram per day

Independent t-test, chi-square test, and Mann Whitney U test (^*^) were used. A *P*-value below 0.05 was considered statistically significant

### Daily dietary intake and physical activity of participants

Table 2 displays the detailed information on dietary intake in two groups. The average daily intake of vegetables and fruits in the insufficient group was 50 g (*P* = 0.022) and 150 g (*P* < 0.001) lower than that of the control group, respectively. The average daily intake of seafood and nuts in the insufficient group was 11.55 g (*P* = 0.001) and 18.5 g (*P* = 0.003) lower than that in the control group. There was no statistical difference in the daily intake of other foods. Table 3 shows the physical activity of participants. There was no statistical difference in the duration of physical activity of each intensity between the two groups. Therefore, physical activity-related variables were not included in the adjustment model.

**Table.2:** Daily dietary intake of participants in the second trimester

Item	All	Soy intake < 40 g/d	Soy intake ≥40 g/d	*P* value
Duration of folic acid intake before pregnancy (median (IQR), month)	3.00 (1.6)	3.00 (3.0)	2.00 (1.4)	0.566
Duration of folic acid intake during pregnancy (median (IQR), month)	4.0 (2.0)	4.00 (2.0)	4.00 (2.0)	0.640
Grains (mean ± SD, g/d) ^*^	359.55 ± 183.73	342.27 ± 172.73	381.37 ± 195.45	0.114
Vegetables (median (IQR), g/d)	300.00 (350.00)	250.00 (300.00)	300.00 (400.00)	0.022
Fruits (median (IQR), g/d)	425.00 (375.00)	350.00 (300.00)	500.00 (450.00)	< 0.001
Meat (median (IQR), g/d)	200.00 (277.80)	172.40 (223.00)	213.00 (284.00)	0.165
Seafood (median (IQR), g/d)	26.75 (47.44)	17.45 (34.69)	29.00 (62.44)	0.001
Eggs (median (IQR), g/d)	50.00 (14.50)	50.00 (14.50)	50.00 (14.50)	0.274
Nuts (median (IQR), g/d)	29.00 (56.50)	25.00 (42.75)	43.50 (85.00)	0.003
Milk (median (IQR), g/d)	250.00 (322.50)	250.00 (322.50)	250.00 (343.13)	0.875
Oils (median (IQR), g/d)	30.00 (45.00)	30.00 (35.00)	30.00 (65.00)	0.719

*Note*: *M* mean, *SD* standard deviation, *IQR* interquartile range, *g/d* gram per day

Independent t-test (^*^) and Mann Whitney U test were used. A *P*-value below 0.05 was considered statistically significant

**Table.3:** Physical activity of participants in the second trimester

Item	All	Soy intake < 40 g/d	Soy intake≥40 g/d	*P* value
Physical activity intensity				
Rest (mean ± SD, 0.9MET ~ 1MET)^*^ Mild (median (IQR), 1.5METs ~ 2METs) Moderate (mean ± SD, 3METs ~ 6METs)* Vigorous (median (IQR), >6METs)	13.71 ± 3.41 7.81 (3.14) 2.54 ± 1.84 0 (0)	13.80 ± 2.67 8.00 (3.14) 2.45 ± 1.79 0 (0)	13.59 ± 2.61 7.69 (3.14) 2.65 ± 2.14 0 (0)	0.556 0.907 0.419 0.907

*Note*: *M* mean, *SD* standard deviation, *IQR* interquartile range, *MET* metabolic equivalent, *1MET* oxygen consumption 3.5 ml/ (kg· min) = energy consumption 0.0167 kcal/ (kg. min), *g/d* gram per day

Independent t-test (^*^) and Mann Whitney U test were used. A *P*-value below 0.05 was considered statistically significant

### Pregnancy outcome of participants

Table 4 displays the maternal and neonatal outcomes. A total of 36 pregnant women were diagnosed with GDM. The incidence of GDM in the insufficient group was 12.5% higher than that in the control group (*χ*^*2*^ = 6.409, *P* = 0.011). The rate of cesarean section in the insufficient group was 14.3% higher than that in the control group (*χ*^*2*^ = 4.370, *P* = 0.037). No significant association was found between soy consumption and macrosomia. Additionally, there was no difference in other maternal and neonatal outcomes.

**Table 4:** Maternal and neonatal outcome

Outcome	All	Soybean intake < 40 g/d	Soybean intake ≥40 g/d	*P*
**Maternal outcome**				
GDM n (%) ^*^	36 (16.1)	27 (21.6)	9 (9.1)	0.011
OGTT fasting glucose (mean ± SD, mmol/L)	4.22 ± 0.36	4.26 ± 0.37	4.17 ± 0.35	0.063
OGTT 1 h glucose (mean ± SD, mmol/L)	7.72 ± 1.81	7.91 ± 1.86	7.48 ± 1.74	0.081
OGTT 2 h glucose (mean ± SD, mmol/L)	6.82 ± 1.38	6.94 ± 1.36	6.68 ± 1.38	0.158
HbA1c (mean ± SD, %)	4.62 ± 0.29	4.65 ± 0.30	4.58 ± 0.27	0.099
Fasting glucose (third trimester, mean ± SD, mmol/L)	4.79 ± 0.95	4.84 ± 0.90	4.73 ± 1.02	0.500
PIH n (%) *	7 (3.1)	4 (3.4)	3 (3.2)	0.999
Delivery gestational age (mean ± SD, weeek)	39.05 ± 1.48	39.00 ± 1.53	39.10 ± 1.42	0.626
Weight gain (mean ± SD, kg)	13.00 ± 4.00	12.88 ± 3.85	13.13 ± 4.18	0.654
Delivery mode n (%) ^*^				0.037
Vaginal delivery	93 (41.5)	44 (37.3)	49 (51.6)	
Cesarean section	120 (53.6)	74 (62.7)	46 (48.4)	
Missing	11 (4.9)	–	–	
Postpartum hemorrhage n (%) ^*^	16 (7.1)	10 (8.5)	6 (6.3)	0.552
Preterm birth n (%) ^*^	17 (7.6)	10 (8.5)	7 (7.4)	0.767
PROM n (%) ^*^	51 (22.8)	25 (21.2)	26 (27.4)	0.293
**Newborn outcome**				
Macrosomia (≥4000 g) n (%) ^*^	9 (4.0)	7 (5.9)	2 (2.1)	0.300
Birth weight (mean ± SD, g)	3231.29 ± 473.83	3225.34 ± 483.07	3238.68 ± 464.55	0.839
Height (mean ± SD, cm)	49.61 ± 2.1	49.53 ± 2.17	49.71 ± 2.02	0.533

Note: *M* mean, *SD* standard deviation, *GDM* gestational diabetes mellitus, *OGTT* oral glucose tolerance test, *PIH* pregnancy-induced hypertension, *PROM* premature rupture of membrane, *g/d* gram per day

Categorical variables (^*^) were described as frequency (percentage). Continuous variables were described as mean (standardization). Independent t-test and chi-square test (^*^) were used. A *P*-value below 0.05 was considered statistically significant

### Multivariate analysis of soy intake and pregnancy outcomes

A multiple logistic regression analysis was performed to explore the association between daily soy consumption and pregnancy outcomes (Table 5). Compared with the daily intake of 40 g or more, a daily intake of 40 g or lower soy was associated with 1.755-fold (95%CI 1.230-6.174, *P* = 0.014) increased risk of developing GDM. After adjusting for age, pre-pregnancy body mass index (BMI), parity, and daily intake of vegetables, fruits, seafood, and nuts, the *OR* was 3.116 (95%CI 1.228-7.907, *P* = 0.017). Compared with the daily 40 g or more of soy intake, consumption of 40 g or less was associated with 0.792-fold (95%CI 1.035-3.101, *P* = 0.037) increased risk of cesarean section. However, there was no significance after adjusting age, pre-pregnancy BMI, parity, and daily intake of vegetables, fruits, seafood, and nuts.

**Table.5:** The association soybean intake and risk of GDM and cesarean delivery

Outcome	*OR* (95%CI) ^a^	*P* value	Adjust *OR* (95%CI) ^b^	*P* value	Adjust *OR* (95%CI) ^c^	*P* value
**GDM**						
Per soybean-unit change	0.992 (0.985-0.999)	0.030	0.993 (0.985-1.001)	0.084	0.992 (0.983-1.000)	0.050
Soybean intake						
≥40 g/d	Reference	–	Reference	–	Reference	–
< 40 g/d	2.755 (1.230-6.174)	0.014	2.653 (1.118-6.296)	0.027	3.116 (1.228-7.907)	0.017
**Cesarean section**						
Per soybean-unit change	0.996 (0.993-1.000)	0.044	0.996 (0.992-1.000)	0.052	0.997 (0.993-1.001)	0.167
Soybean intake						
≥40 g/d	Reference	–	Reference	–	Reference	–
< 40 g/d	1.792 (1.035-3.101)	0.037	1.665 (0.949-2.924)	0.076	1.421 (0.772-2.617)	0.259

*GDM* gestational diabetes mellitus, *OR* odds ratio

^a^ unadjusted

^b^ After adjusted for age, pre-pregnancy body mass index, and parity (nulliparous/multiparous)

^c^ After adjusted for age, pre-pregnancy body mass index, parity (nulliparous/multiparous), daily intake of vegetables, fruits, seafood, and nuts

Logistic regression analysis method was used. A *P*-value below 0.05 was considered statistically significant

## Discussion

The present cohort study evaluated the association of soy intake with the risk of GDM and cesarean section. We found that more than half of pregnant women consumed less soy than the recommended amount, and daily soy intake of less than 40 g was associated with the increased risk of GDM. Even after adjusting for confounders such as age, pre-pregnancy BMI, parity, daily intake of vegetables, fruits, seafood, and nuts, daily soy intake of less than 40 g increased 2.116-fold risk of GDM. Additionally, there was no significant difference in the increased risk of cesarean sections with soy intake below 40 g/d after adjustment for confounding factors. Therefore, we assumed that the correlation between soy consumption and cesarean section risk reduction was merely due to confounders and not due to the soy consumption itself. Our findings suggested that adequate soy intake may have a beneficial role in the prevention of GDM.

This study found that more than half (55.8%) of pregnant women consumed soy less than the recommended amount [24], indicating the situation of Chinese pregnant women’s soy intake was not optimistic. Our previous study [18] revealed that the daily soy intake of urban pregnant women in the early, middle, and late trimesters was 19.10 g, 19.42 g, and 21.50 g, respectively, less than half of the recommended value. This situation was even worse in rural pregnant women living in the poverty-stricken area, which revealed only 8.98% of pregnant women eat soy or soy products every day, 19.18% of pregnant women never eat soy or soy products during pregnancy [25]. A survey conducted by Huang in Guangzhou showed that 9.9% of women consumed soy and soy products every day during pregnancy, and only 6.50 g/day of soy was consumed in the third trimester [26]. These findings were also supported by studies in other areas in China, such as Urumqi [27], Xiamen [28], Lanzhou [19], and Anhui [29]. Although health care providers inform pregnant women of the need to consume sufficient soy during pregnancy, pregnant women are not following the guidance well. This may be because pregnant women are not aware of the importance of soy intake for healthy outcomes, such as improving metabolic profiles [14] and decreasing the risk of preterm birth and maternal anemia in late gestation [10]. In addition, there may be external barriers such as difficulty in obtaining soy, poor taste of soy, bloating, and other uncomfortable reactions after ingesting soy, etc. Therefore, health care providers should make pregnant women be aware of the importance and benefits of soy intake, and introduced soy alternatives such as tofu and soy milk. The intake amount of soy products varies according to different processing methods and levels because different soy products have different amounts of protein and other components. For example, 100 g of soybeans contains 35.1 g of protein, 100 g of soybean milk and soybean milk powder contain 1.8 and 19.7 g of protein, respectively and 100 g of tofu related products, such as lactone tofu, dried tofu and shredded tofu, contain between 5 and 57.8 g of protein [30]. More importantly, health care providers should also recommend soy intake for pregnant women following local dietary guidelines and advise them not to exceed the maximal intake limit recommendation. Soy should not be recommended for pregnant women who are allergic to it. Additionally, soy consumption should be cautiously recommended for pregnant women with kidney disease, severe stomach ulcers, gout, and cardiovascular disease.

Our findings agreed with one previous cohort study, which surveyed the dietary of 1,129 Chinese pregnant women and found the risk of GDM in pregnant women decreased gradually with the gradual increase of the daily intake of total legumes and the daily intake of soy and soy products. The inverse association was particularly significant in pregnant women with normal pre-pregnancy BMI and no family history of diabetes [12]. These findings were similar to Goshtasebi’s study [31], which prospectively recruited 1029 Tehranian pregnant women and found that legumes consumption ≥3.3 servings/week decreased 0.62-fold risk of GDM. A large cohort study conducted in Japan found the intakes of isoflavones, genistein, miso soup, and natto were inversely associated with the incidences of GDM [13]. Many studies based on Asian and Middle Eastern populations have confirmed the association between insufficient soy intake and the risk of GDM [12, 13, 31]. However, few studies examined the relationship between soy intake and GDM based on European, American, and other populations. Some studies performed in Europe and Mediterranean countries have explored maternal dietary patterns and risk of GDM and found dietary patterns with a higher intake of fruits, vegetables, legumes, whole grains, and fish are associated with a decreased likelihood of GDM [32–34]. Current evidence shows that adequate soy intake may prevent the occurrence of GDM. A further prospective randomized controlled trial is needed to verify the preventive effect. Additionally, further research is of interest in understanding more clearly which product and ingredient of soy play a beneficial role in preventing GDM.

The mechanisms of the negative association between soy intake and risk of GDM remain unclear. Only one interventional study compared the effects of soy treatment and control diet on metabolic profiles of women with GDM and found that soy protein consumption improved the glucose homeostasis parameters, triglycerides, and biomarkers of oxidative stress such as total antioxidant capacity and glutathione [14]. Another interventional study found soybean oligosaccharides were able to reduce oxidative stress and alleviate insulin resistance of women with GDM [35]. Although there is still a lack of research on the mechanism of soy and GDM, studies on the relationship between soy intake and type 2 diabetes and glucose metabolism may provide references. Many studies have identified the antidiabetic effects of soy [1, 8, 36]. Soy contains large amounts of flavonoids with antioxidant and anti-inflammatory properties, and high amounts of minerals, which play a beneficial role in controlling blood glucose and glucose homeostasis, respectively [37, 38]. Soy isoflavones and protein are the two main functional components of soy and soy products such as tofu and soy milk, which are related to blood glucose metabolism. A meta-analysis reported that soy isoflavone supplementation could be beneficial for body weight reduction, glucose, and insulin control in plasma [39]. Moreover, soy isoflavones play a beneficial role in rebelling insulin resistance by increasing glucose transporter-4 levels, down-regulating peroxisome proliferator-activated receptor-γ, and increasing short-chain fatty acid-producing bacteria in the gut [40]. Genistein is generally considered as the one of major active ingredients of soy isoflavones, which may be associated with diabetes. Genistein has positive effects on the survival, the proliferation of pancreatic β cells and can act directly on pancreatic β cells, and consequently increase insulin secretion [4]. Genistein also has insulinotropic effects by activating cAMP/protein kinase A signaling cascade [4]. Additionally, genistein plays a role in improving oxidative stress-induced injury and affecting the concentration of individual plasma lipids, adiponectin, and other cytokines [41], which are related to the occurrence and development of GDM. Similarly, soy protein is shown to have favorable effects on fasting blood glucose, insulin, HOMA-IR, and triglycerides [14] and also verified to increase gene expression of peroxisome proliferator-activated receptors, which involved in the pathogenesis of GDM [42, 43]. In addition, soy protein and isoflavones may influence diabetes-related cytokines such as TNF-alpha and IL-1 [44], then improve glucose metabolism. Based on the evidence from the above studies, soy can improve insulin resistance, pancreatic β-cell dysfunction, oxidative stress, and inflammatory response, etc. of the human body, thus contributing to GDM risk reduction.

This study had several limitations. First, a semi-quantitative FFQ was used in the study. This dietary assessment tool was not an accurate one compared with the weighing method. Additionally, there was recall bias in the FFQ survey, which may have an impact on results. However, FFQ is a simple dietary assessment tool and widely used in many studies. The Cronbach coefficient of FFQ showed good internal consistency. Therefore, we considered that FFQ recall bias does not have a significant impact on the results of this study. Second, some food intake that may affect glucose homeostasis such as sweets, snacks, and sweetened beverages were not evaluated. As an observational study, we could not rule out the influence of confounding. Although we carefully controlled for known risk factors and potential confounding factors, our results may be affected by other unmeasured factors related to soy intakes. Third, IADPSG criteria were used in this study, which resulted in a higher incidence rate of GDM compared with that in other populations. This may be explained by the fact that the positive detection rate of GDM of two-hours 75-g OGTT was higher than that of 100-g OGTT. Additionally, we didn’t investigate the type and matrix components of soy products and method processing of soy, so we were unable to analyze whether these factors have a potential impact on the relationship between soy and GDM.

## Conclusions

In conclusion, this study found that more than half of pregnant women consumed less soy than 40 g per day. After adjusting for confounders such as age, pre-pregnancy BMI, parity, daily intake of vegetables, fruits, seafood, and nuts, daily soy intake of less than 40 g increased risk of GDM, but not with the increased risk of cesarean section. It suggested that insufficient soy intake increased the incidence rate of GDM. Pregnant women without clear contraindications should follow dietary guidelines to consume an appropriate amount of soy. It was important to note that the intake amount of soy products should vary according to different processing methods and levels. A further randomized controlled trial should be conducted to verify this preventive effect and its mechanisms, and which product and ingredient of soy play a beneficial role in preventing GDM should be cleared.

## Data Availability

The datasets used and/or analyzed during the current study are available from the corresponding author on reasonable request.

## Abbreviations

GDM: Gestational diabetes mellitus
M ± SD: Mean ± Standard Deviation
OGTT: Oral glucose tolerance test
FFQ: Food Frequency Questionnaire
PAS: Physical Activity Scale
HOMA-IR: Homeostasis model assessment of insulin resistance
BMI: Body mass index
MET: Metabolic equivalent
